# Objectively measured versus self-reported occupational physical activity and multisite musculoskeletal pain: a prospective follow-up study at 20 nursing homes in Denmark

**DOI:** 10.1007/s00420-019-01495-4

**Published:** 2019-11-23

**Authors:** Subas Neupane, Kristina Karstad, David M. Hallman, Reiner Rugulies, Andreas Holtermann

**Affiliations:** 1 Unit of Health Sciences, Faculty of Social Sciences, Tampere University, 33014 Tampere, Finland; 2 Gerontology Research Center, Tampere University, 33014 Tampere, Finland; 3 Finnish Institute of Occupational Health, Helsinki, Finland; 4 National Research Centre for the Working Environment, Copenhagen, Denmark; 5 Department of Occupational Health Sciences and Psychology, Centre for Musculoskeletal Research, University of Gävle, Gävle, Sweden; 6 Department of Psychology, University of Copenhagen, Copenhagen, Denmark; 7 Department of Public Health, University of Copenhagen, Copenhagen, Denmark

**Keywords:** Musculoskeletal pain, Multisite pain, Occupational physical activity, Objective measures, Self-report

## Abstract

**Purpose:**

To explore the prospective association of objectively measured and self-reported occupational physical activity (OPA) with multisite musculoskeletal pain (MSP) among Danish eldercare workers.

**Methods:**

The study population consisted of eldercare workers in 20 Danish nursing homes (*N* = 553, response rate 59%, 525 female). Baseline data were collected in 2013–2014 and the 1-year follow-up was completed in 2016. At baseline, we measured objective OPA by a thigh-worn ActiGraph GT3X + accelerometer during work and self-reported OPA by a questionnaire survey. Information on musculoskeletal pain during the past four weeks in seven different body sites was reported by a structured questionnaire at baseline (*n* = 389) and by SMS and telephone interview during follow-up (*n* = 284). MSP was defined as having pain in two or more body sites. Using log-binomial models we calculated risk ratios (RRs) with their 95% confidence intervals (CIs) to estimate the association between objectively measured and self-reported OPA and MSP.

**Results:**

We found statistically significant positive associations between self-reported OPA (RR for high OPA 1.24, 95% CI 1.05–1.46) and MSP while there was no significant association found between objective OPA and MSP.

**Conclusion:**

Our study indicates that self-reported, but not objectively measured OPA is positively associated with MSP. This finding highlights the need for better understanding, use, and interpretation of self-reported and objectively measured OPA in the study of MSP.

## Introduction

Musculoskeletal pain is common in the general and working population (Vos et al. 2017) and affects both working careers and life after retirement (WHO 2003). Musculoskeletal pain commonly occurs in more than one part of the body (Carnes et al. 2007). Multisite musculoskeletal pain (MSP) appears relatively stable over time (Kamaleri et al. 2009; Neupane et al. 2018), with studies of working age to older age people showing diminishing pain after retirement (Neupane et al. 2018). Earlier studies have reported that compared to single-site pain, MSP has severe consequences for daily functioning (Saastamoinen et al. 2006; Kamaleri et al. 2008) poor work ability and sickness absence (Neupane et al. 2015; Haukka et al. 2006).

Despite the negative impact of MSP on the working population, risk factors of MSP have not been established. Earlier studies have reported several occupational exposures (Neupane et al. 2018; Kamaleri et al. 2008; Haukka et al. 2011) and lifestyle factors (Kamaleri et al. 2008) as determinants of MSP. Physical exposures at work such as lifting heavy loads, bending or twisting, vibrations, awkward postures and biomechanical loads (Haukka et al. 2011; Neupane et al. 2013; Herin et al. 2014; Coggon et al. 2013; Lourenço et al. 2015psychosocial exposures such as job demands, co-workers support, and somatization (Solidaki et al. 2010) have been reported as predictors of MSP. It is conceivable that more favourable workplace factors might reduce the risk of MSP and alleviate the burden of MSP in the working population.

While some studies have reported a negative association between objectively measured physical activity and MSP (Pan et al. 2019), these did not specifically address working populations and did not examine physical activity at work. Another study (Murata et al. 2018) reported lower objectively measured physical activity associated with the number of pain sites among older adult in community-dwelling. However, these studies have not distinguished occupational physical activity from total or leisure time. We expect that both the objective and self-reported occupational physical activity (OPA) would have a same non-beneficial effect on MSP.

Most previous evidence on the associations between workplace factors and MSP is based on self-reported physical exposures at work, which are susceptible to bias (Prince et al. 2008). Earlier studies showed that individuals usually under- or over-estimate their physical activities. For example, the total physical activity level was overestimated by 36–173% (Lee et al. 2011). The bias related to self-reported occupational physical activity may be differential and may lead to an overestimation of the association between self-reported OPA and MSP. Gupta et al. (Gupta et al. 2018) reported that the extent of overestimating self-reported physical activity time depended on the level of musculoskeletal pain. Those with a high level of musculoskeletal pain overestimated their physical activity to a higher extent than those with a low level of musculoskeletal pain.

To our knowledge, no earlier studies have examined the association between objectively measured occupational physical activity and MSP. Further, no earlier studies have investigated if the direction and strengths of the association between occupational physical activity and MSP depend on whether physical activity was measured objectively or by self-report. In this study, we, therefore, aimed to explore the association between both objectively measured OPA and self-reported OPA and MSP among Danish eldercare workers.

## Methods

**Fig. 1:**
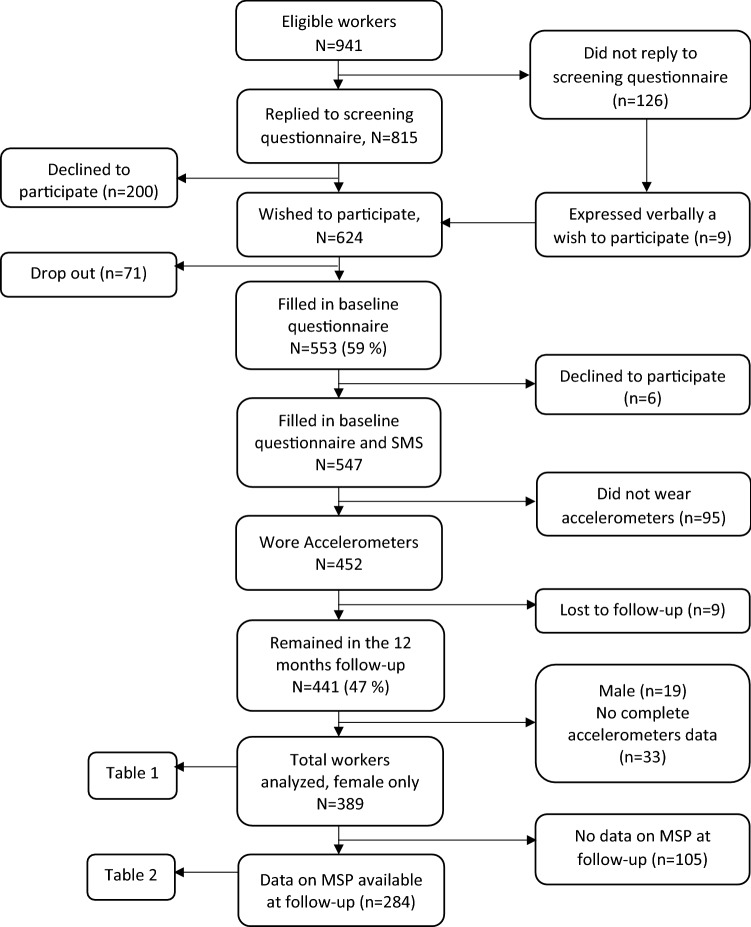
Flow diagram of the study participation

This study is a part of a prospective study of Danish Observational Study of Eldercare work and musculoskeletal disorderS (DOSES). The study protocol with data collection, design and methodology has been described in detail elsewhere (Karstad et al. 2018). In brief, this study was conducted among eldercare workers aged between 18 and 65 years, employed in Danish nursing homes, and working more than 15 h per week on the day and/or evening shifts. We excluded individuals that were on long-term sickness absence, pregnant, not permanently employed and not working a minimum of 25% of their working time on task related to the direct care of the elderly. In total, 83 nursing homes located in the Copenhagen area were invited to participate in the study and 20 nursing homes (18 municipal and 2 private nursing homes) with an average of 70 eldercare workers agreed to participate in the study (Karstad et al. 2018). A short screening questionnaire including a question on whether the eldercare workers would like to participate in the study was administered to 941 eligible eldercare workers. Those who wished to participate were invited to the questionnaire survey, accelerometer measurement, health, and physical capacity measurement. In total, *N* = 553 (response rate 59%, 525 female) replied to the baseline questionnaire. Those that participated at baseline were followed-up after 1 year by text messages and telephone interview to which 441 workers responded (Fig. 1). However, during follow-up, we analyzed only the participants who had information on OPA and musculoskeletal pain (*n* = 284). The baseline data were collected from September 2013 to December 2014 and the follow-up completed in January 2016. Data on sociodemographic characteristics, lifestyle, health, and work-related factors were collected using computer-based structured questionnaire when participants attended a 45 min long health check session held at their workplace.

Individuals provided informed consent to participate in the study when they came for the individual measurements. The Danish Data Protection Agency and the Ethics Committee for the Capital Region of Denmark provided the ethical approval to this study.

### Measurement of occupational physical activity

#### Objective measurements

Physical activity (e.g. walking, running and cycling), body postures (e.g. sitting and standing) and movements (e.g. forward bending of the back and arm elevation) both at work and leisure time was measured using ActiGraph GT3X+ accelerometers (ActiGraph, Florida, United States) at baseline. Participants were asked to wear three accelerometers each on the thigh, upper back and dominant arm for a minimum of 4 consecutive days including at least 2 working days. Those with an allergy to patches were exempted from wearing the accelerometers and consequently excluded from the study. Acti4, a validated software program with high sensitivity and specificity (Skotte et al. 2014; Stemland et al. 2015), was used to analyze the accelerometer data.

In total, 452 participants wore the accelerometers and provided the data. For the analyses in this study, we included only female participants, as only a few men (< 5%) participated in the study, who had at least 2 days of measurements of physical activity via accelerometer placed at the thigh (*N* = 389). We excluded all non-working days, sleep periods, and non-wear periods from the analysis. The participants had on average 17 h accelerometer (thigh) wear time per day (time with measurements of physical activity). On average, the participants had a total of 17 h of accelerometer wear time during work.

Objectively measured occupational physical activity, (OPA), was measured at baseline as the sum of the cycling, moving, rowing, and running, climbing stairs, standing and walking activities at work in an hour/day. We used both continuous score and categorical OPA in the analysis. A continuous score of OPA was used to check the consistency of the associations when using categorical information. Tertiles values were used as a cut-off point to create three categories (low, medium and high) of OPA.

#### Self-reported measurements

Self-reported occupational physical activity was measured in terms of physical demands at baseline with the question “how physically demanding do you normally consider your present work?” and the response in a scale of 0–10 (0 = not strenuous, 10 = maximum strenuous). The response was categorized into three categories (low, medium and high) for the analysis using tertiles as cut-off points.

### Multisite musculoskeletal pain (MSP)

Musculoskeletal pain experienced during the past 4 weeks in seven different locations (neck/shoulder, lower back, elbows, hands/wrist, hips, knees and feet/ankles) of the body was measured in a scale of 0-10 (0 = no pain, 10 = worst imaginable pain). At baseline, the information on musculoskeletal pain was collected using a structured questionnaire, while at 1-year follow-up the information was collected using SMS and telephone interview. At follow-up, the information on neck/shoulder and lower back pain were collected with SMS and the rest of the body-sites with telephone interview. The response by SMS was slightly higher than for the telephone interview. Each of the responses for pain in the different body locations was first dichotomized (no/yes) by using median value as cut-off points (median and lower = 0, higher than median = 1) (Neupane et al. 2013). The dichotomized responses were then summed up and formed a score 0–7 where 0 indicates no pain in any of the body sites and 7 indicates pain in all seven body sites. The score 0–7 were further categorized into two; 0 and 1 combined as no multisite pain and 2–7 was combined as multisite pain.

### Covariates

Baseline information on two psychosocial variables at work; *influence at work* and *social support* was obtained. These psychosocial variables were included because they were related to the MSP in previous studies (Kamaleri et al. 2008; Solidaki et al. 2010). *Influence at work* was measured using two questions on the degree of influencing own work and the amount of work on a scale of 1–5 (1 = always, 5 = never). These two items were then summed up and dichotomized (low and high influence) using median value as a cut-off point. Similarly, *social support* was measured using four items on the support obtained from coworkers for e.g. practical help, advice, and guidance, listening to problems and talking about work on a scale of 1–5 (1 = always, 5 = never). The sum scale was then dichotomized (low and high social support) using median value as the cut-off.

Demographic information included age in years, gender, job seniority in years, and the workplace were obtained at baseline from the questionnaire. Height and weight measurement were taken during the health check. Body mass index (BMI) was calculated as the weight divided by the square of the height in the unit kg/m^2^. Information on smoking was obtained by a questionnaire with three response category (1 = yes, daily, 2 = yes, sometimes, 3 = previously smoking, 4 = no, never). In this analysis option, 1 and 2 were combined as smokers. Covariates were selected based on earlier literature as well as their availability in the data set.

### Statistical analysis

We first calculated the distribution of baseline demographic, lifestyle, work-related characteristics and multisite musculoskeletal pain of the studied participants stratified by three categories of objective OPA to show the difference between three categories of objective OPA. Mean and standard deviation was calculated for the continuous variables while the frequency and the percentages were calculated for the categorical variables. The *p* value for the difference in the distribution between three categories of objective OPA was tested using Chi-square test for the categorical variables and ANOVA test for the continuous variables.

The longitudinal association of MSP with both the OPA variables was analyzed using log-binomial models. Risk ratios (RRs) and their 95% CIs were presented as the measure of longitudinal associations. Log-binomial models are similar to logistic regression models, but add a log link function to connect the dichotomous outcome to the linear predictor. Log-binomial models are used to directly estimate risk ratios in prospective studies for both common and rare outcomes (Zochetti et al. 1995). Models were built in two steps: Model I was univariate association and Model II was adjusted for age, job seniority, type of workplace, smoking and BMI and psychosocial factors (influence at work and social support) and baseline multisite pain.

All analysis was conducted in STATA version 14.

## Results

### Study participants’ characteristics at baseline

Table 1 shows the baseline characteristics of the study participants stratified by objectively-measured OPA. The mean age of the participants was 46.3 ± 10.5 years and there was a significant difference in the mean age between three groups of objective OPA with youngest in low objective OPA group and oldest in high OPA group. BMI distribution was statistical significantly different between objective OPA groups with higher mean BMI (27.7 kg/m^2^) in low OPA compared with medium OPA (BMI 25.6 kg/m^2^) and high OPA (BMI 25.8 kg/m^2^). Among work-related factors, statistically significant difference between objective OPA groups with a continuous score of objective OPA was found. The interquartile range of objective OPA was 0.94–1.40 h with the mean value 1.16 h. No statistical significant difference between the objective OPA groups was found for self-reported OPA. However, those with medium to high OPA also reported high self-reported OPA. The interquartile range of self-reported OPA (original continuous scale) was 6–8 and the mean value 6.84 in a scale of 0-10. Perceived influence and social support at work differed significantly between objective OPA groups with the highest influence and social support among those with high OPA. There was no statistically significant difference in the prevalence of MSP among workers between objective OPA groups, but comparatively more workers in low OPA group reported MSP. The prevalence of MSP was 74% (*n* = 285) at baseline and 70% (*n* = 200) at follow up. Among those with MSP at baseline, 80% also had MSP at follow-up. The most common pain sites were neck shoulder and low back pain both at baseline (neck-shoulder 54%, low back 54%) and at follow-up (neck-shoulder 59%, low back 58%), while elbow pain had the lowest prevalence both at baseline (17%) and at the follow-up (16%).

**Table 1:** Baseline characteristics of the study population stratified by objectively measured occupational physical activity (OPA) (in hours) among women

Characteristics	Total *N* = 389	Objective OPA^a^			
		Low	Medium	High	*p* value
Age in years, mean ± SD	46.3 ± 10.5	43.6 ± 10.3	45.4 ± 10.0	49.9 ± 10.2	< 0.001
Job seniority in years, mean ± SD	18.2 ± 11.2	17.7 ± 10.1	17.6 ± 11.3	19.1 ± 12.1	0.509
BMI in kg/m^2^, mean ± SD	26.4 ± 5.0	27.7 ± 5.7	25.6 ± 5.1	25.8 ± 4.8	0.001
Smokers, *n* (%)	140	42 (30.0)	53 (37.9)	45 (32.1)	0.288
Work-related factors					
Objective OPA, mean ± SD	1.16 ± 0.36	0.79 ± 0.22	1.17 ± 0.09	1.56 ± 0.18	< 0.001
Self-reported OPA, *n* (%)					0.101
Low	142	60 (42.2)	39 (27.5)	43 (30.3)	
Medium	164	49 (29.9)	59 (36.0)	56 (34.1)	
High	82	22 (26.8)	31 (37.8)	29 (35.4)	
Influence at work, *n* (%)					0.005
Low	266	104 (39.1)	84 (31.6)	78 (29.3)	
High	123	28 (22.8)	45 (36.6)	50 (40.6)	
Social support, *n* (%)					0.047
Low	212	82 (38.7)	70 (33.0)	60 (28.3)	
High	177	50 (28.3)	59 (33.3)	68 (38.4)	
Baseline multisite pain, *n* (%)					0.869
No	101	33 (32.7)	35 (34.6)	33 (32.7)	
Yes	285	99 (34.7)	93 (32.7)	93 (32.6)	

^a^Objective OPA was measured as a sum of cycling, running, stairs climbing and walking

### Associations between occupational physical activity and multisite pain

Table 2 shows the longitudinal association of MSP with objectively measured and self-reported OPA. Although not statistically significant, a continuous score of objective OPA was associated with MSP in the final model with a 10% increased risk per unit increased in objective OPA. We found no association of high objective OPA with MSP.

**Table 2:** Longitudinal association of multisite musculoskeletal pain (MSP) with objective OPA and self-reported OPA

Characteristics	MSP^a^*n* = 200	RR, 95% CI			
		Model I	*p* value	Model II	*p* value
Objective OPA (continuous)		1.10 (0.88–1.36)	0.410	1.10 (0.88–1.37)	0.394
Objective OPA					
Low	63	1.0		1.0	
Medium	73	1.13 (0.94–1.37)	0.200	1.12 (0.94–1.35)	0.210
High	64	1.11 (0.91–1.34)	0.304	1.00 (0.85–1.20)	0.937
Self-reported OPA					
Low	66	1.0		1.0	
Medium	83	1.18 (0.98–1.43)	0.084	1.13 (0.98–1.29)	0.088
High	50	1.36 (1.13–1.65)	0.001	1.24 (1.05–1.46)	0.011

Risk ratios (RRs) and 95% confidence intervals (CIs) for MSP from log-binomial regression model (*N* = 284)

Model I: Univariate association

Model II: Adjusted for age, job seniority, type of workplace, smoking, BMI, influence at work, social support and baseline multisite pain

^a^Those reported multisite pain at follow-up (*n* = 200)

High self-reported OPA was statistical significantly associated with MSP. The magnitude of the association was stronger in the crude model, but the association remained statistically significant when the models were adjusted for age, job seniority, type of workplace, smoking, BMI, influence at work, social support and baseline multisite pain (RR 1.24, 95% CI 1.05–1.46) in Model II.

## Discussion

### Summary of results

We found in this prospective follow-up study among eldercare workers that self-reported high OPA was associated with MSP, while high objectively measured OPA was not significantly associated with MSP. The central finding is that the association between OPA and MSP seems to depend on whether OPA is measured by self-report or in an objective way.

### Interpretation

To the best of our knowledge, this is the first study examining the association of MSP with objectively measured OPA in a prospective follow-up design. Although not statistically significant, we found a positive association of objectively measured OPA with MSP. Several explanations maybe suggested for the no statistically significant association between objective OPA and MSP. First, the exposure levels of occupational activities in this study may not lead to increased risk of MSP over 1 year. Second, the OPA measure is a composite measure of exposures to many physical activities, which might then not grasp potential specific activities providing a sign of risk. Third, the OPA measure does not incorporate heavy manual handling of time-pattern including breaks. It is also possible that we did not capture relevant OPAs in the objective measurement which self-reported OPA may have captured for e.g. intensities of manual activities compared to objectively measured time spent in walking/running. It is also possible that the accelerometer set up on the thigh and upper back did not measure lower back movement (such as forward bending) which is probably reflected in the self-reports. Moreover, self-reported OPA likely reflects the balance between the capacity of the worker and the actual exposure to OPA (Merkus et al. 2019), while the objective measurement provides data on absolute exposure levels. However, objective OPA in our study was the sum measures of cycling, moving, rowing, running, climbing stairs, standing and walking activities at work which possibly captured physical activities at work for a typical working day (Loef et al. 2018). OPA was detected from processed accelerometer signals which is a valid and accurate measure of physical activities at work (Skotte et al. 2014; Stemland et al. 2015) and have been used in previous studies (Loef et al. 2018; Hallman et al. 2017).

The healthy-worker effect may also have affected the result in this, as well as in any, prospective workplace study. It may have leveled off the exposure differences through the selection of workers with pain to tasks with lower objective OPA or out of the workforce as there were comparatively more workers in the medium OPA group who had MSP at the follow-up. Over-estimating of self-reported physical activities at work may also have led to the difference in the association of the objective and self-reported physical activities with MSP in our study. Nevertheless, the strong association of self-reported OPA with MSP suggest that this explanation of no significant association of objective OPA with MSP is less likely.

Although not statistically significant, workers with medium OPA had higher risk of MSP than those exposed to low objective OPA. This could be because medium OPA is related to other biomechanical exposures that could influence MSP.

### Comparison with previous studies

Consistent with previous studies (Coggon et al. 2013; Neupane et al. 2017; Haukka et al. 2012; Madsen et al. 2018) we found a rather strong positive association between self-reported high OPA and MSP. Eldercare workers are regularly exposed to manual handling activities such as lifting, pulling, and pushing activities, in an awkward body position, which have some causal relationship with musculoskeletal pain (da Costa and Vieira 2010). However, the mechanism behind the role of high OPA on MSP is still not that clear. Moreover, participants with MSP may perceive high OPA which is also a nature of self-reported data and are highly susceptible to bias (Prince et al. 2008). Self-report may not accurately capture the aspect related to time spent in OPA, but perceived demands may contain useful information such as intensities of demanding work not captured by objective measurements used to predict MSP. However, we cannot rule out that the measurement for self-reported OPA, i.e. physical demands was insufficient to capture all physical aspects of work. Nevertheless, the perceptions of self-reported physical demands may also contain unique, important information of predictive value for musculoskeletal outcome since it reflects a combination of load, workers capacity, and a perceptual component of the load (Christensen and Knardahl 2010). Thus, future studies with objective and self-reported measures of OPA, adding more potential confounders with statistical power and probably with longer follow-up are needed to further confirm these findings of our study.

In our study, MSP was very common among eldercare workers. The prevalence of MSP was 74%, and of those who had MSP at baseline about 80% had persistent nature of MSP (i.e. also reporting MSP at follow-up). The results of our study support the findings from earlier studies from Europe and elsewhere which show that MSP is common in working population, i.e. reported week to year prevalence rates from 40 to 73% (Kamaleri et al. 2009; Haukka et al. 2006; Coggon et al. 2013; Solidaki et al. 2010; Neupane et al. 2011, 2016).

### Strengths and limitations

Our study has several strengths. One of the strengths is that we used both objectively measured and self-reported exposure data on occupational physical activity to compare their separate effect on the MSP outcome in prospective follow-up design. Objective measurements are accurate and avoid common methodology bias in observational research (Prince et al. 2008). Our objective measurement covered a typical working day removing all non-working days, sleep periods, and non-wear periods. This study includes sampled data from 20 nursing homes which represent a broad range of nursing homes in Denmark (Karstad et al. 2018). The response rate was reasonable, 59% of the all eligible participants participated in the study. However, we included only participants for whom the objective measurements data and the information on the outcome were available. And, only the female participants were used in our analysis as there were very few male workers. We used both a continuous score of OPA and a categorical. Tertile values were used to determine the cut-off point for the categories of self-reported and objectively measured OPA, which allowed us to have enough cases in all three categories. Although categories may have led to the loss of information to some extent, they are useful for exploring potential non-linear relationships. One of the limitations is that we have included only a single item measure for self-reported OPA (measured in terms of physical demand), while objective OPA was the sum of cycling, running, stairs climbing and walking. Self-reported OPA may not capture all aspects of demanding posture as well as carrying loads and repetitiveness of the work. Nevertheless, the single-item measure of physical demand has been used in predicting musculoskeletal pain and sick leaves in earlier studies (Petersen et al. 2019; Andersen et al. 2016). This study analyzed only women working in nursing homes and therefore the results may not be generalizable to other populations.

In conclusion, our study indicates that self-reported, but not objectively measured OPA, is associated with MSP, which suggests that this relationship depends on whether OPA is measure by self-report or objective measurements. This could be because of the different content of the self-reported and objective OPA. We found a high prevalence of MSP among eldercare workers and the majority of them had a continuation of MSP. Having MSP was associated with baseline high self-reported OPA, while the association with baseline objectively measured OPA was not very clear. This finding highlights the need for better understanding, use, and interpretation of self-reported and objectively measured OPA in the study of musculoskeletal pain.

## References

[CR1] Andersen LL, Fallentin N, Thorsen SV, Holtermann A (2016). Physical workload and risk of long-term sickness absence in the general working population and among blue-collar workers: prospective cohort study with register follow-up. Occup Environ Med.

[CR2] Carnes D, Parsons S, Ashby D, Breen A, Foster NE, Pincus T, Vogel S, Underwood M (2007). Chronic musculoskeletal pain rarely presents in a single body site: results from a UK population study. Rheumatology.

[CR3] Christensen JO, Knardahl S (2010). Work and neck pain: a prospective study of psychological, social, and mechanical risk factors. Pain.

[CR4] Coggon D, Ntani G, Palmer KT, Felli VE, Harari R, Barrero LH, Felknor SA, Gimeno D, Cattrell A, Vargas-Prada S, Bonzini M (2013). Patterns of multisite pain and associations with risk factors. PAIN®.

[CR5] da Costa BR, Vieira ER (2010). Risk factors for work-related musculoskeletal disorders: a systematic review of recent longitudinal studies. Am J Ind Med.

[CR6] Gupta N, Heiden M, Mathiassen SE, Holtermann A (2018). Is self-reported time spent sedentary and in physical activity differentially biased by age, gender, body mass index and low-back pain?. Scand J Work Environ Health.

[CR7] Hallman DM, Birk Jørgensen M, Holtermann A (2017). Objectively measured physical activity and 12-month trajectories of neck–shoulder pain in workers: a prospective study in DPHACTO. Scand J Public Health.

[CR8] Haukka E, Leino-Arjas P, Solovieva S, Ranta R, Viikari-Juntura E, Riihimäki H (2006). Co-occurrence of musculoskeletal pain among female kitchen workers. Int Arch Occup Environ Health.

[CR9] Haukka E, Leino-Arjas P, Ojajärvi A, Takala EP, Viikari-Juntura E, Riihimäki H (2011). Mental stress and psychosocial factors at work in relation to multisite musculoskeletal pain: a longitudinal study of kitchen workers. Eur J Pain.

[CR10] Haukka E, Ojajärvi A, Takala EP, Viikari-Juntura E, Leino-Arjas P (2012). Physical workload, leisure-time physical activity, obesity and smoking as predictors of multisite musculoskeletal pain. A 2-year prospective study of kitchen workers. Occup Environ Med.

[CR11] Herin F, Vezina M, Thaon I, Soulat J-M, Paris C, ESTEV Group (2014). Predictive risk factors for chronic regional and multisite musculoskeletal pain: a 5-year prospective study in a working population. PAIN®.

[CR12] Kamaleri Y, Natvig B, Ihlebaek CM, Benth JS, Bruusgaard D (2008). Number of pain sites is associated with demographic, lifestyle, and health-related factors in the general population. Eur J Pain.

[CR13] Kamaleri Y, Natvig B, Ihlebaek CM, Bruusgaard D (2009). Does the number of musculoskeletal pain sites predict work disability? A 14-year prospective study. Eur J Pain.

[CR14] Karstad K, Jørgensen AF, Greiner BA, Burdorf A, Søgaard K, Rugulies R, Holtermann A (2018). Danish Observational Study of Eldercare Work and musculoskeletal disorderS (DOSES): a prospective study at 20 nursing homes in Denmark. BMJ Open.

[CR15] Lee PH, Macfarlane DJ, Lam TH, Stewart SM (2011). Validity of the international physical activity questionnaire short form (IPAQ-SF): a systematic review. Int J Behav Nutr Phys Act.

[CR16] Loef B, van der Beek A, Holtermann A, Hulsegge G, van Baarle D, Proper KI (2018). Objectively measured physical activity of hospital shift workers. Scand J Work Environ Health.

[CR17] Lourenço S, Araújo F, Severo M, Cunha Miranda L, Carnide F, Lucas R (2015). Patterns of biomechanical demands are associated with musculoskeletal pain in the beginning of professional life: a population-based study. Scand J Work Environ Health.

[CR18] Madsen IE, Gupta N, Budtz-Jørgensen E, Bonde JP, Framke E, Flachs EM, Petersen SB, Svane-Petersen AC, Holtermann A, Rugulies R (2018). Physical work demands and psychosocial working conditions as predictors of musculoskeletal pain: a cohort study comparing self-reported and job exposure matrix measurements. Occup Environ Med.

[CR19] Merkus SL, Lunde LK, Koch M, Wærsted M, Knardahl S, Veiersted KB (2019). Physical capacity, occupational physical demands, and relative physical strain of older employees in construction and healthcare. Int Arch Occup Environ Health.

[CR20] Murata S, Doi T, Sawa R, Nakamura R, Isa T, Ebina A, Kondo Y, Tsuboi Y, Torizawa K, Fukuta A, Ono R (2018). Association between objectively measured physical activity and the number of chronic musculoskeletal pain sites in community-dwelling older adults. Pain Med.

[CR21] Neupane S, Miranda H, Virtanen P, Siukola A, Nygård CH (2011). Multi-site pain and work ability among an industrial population. Occup Med.

[CR22] Neupane S, Miranda H, Virtanen P, Siukola A, Nygård C-H (2013). Do physical or psychosocial factors at work predict multi-site musculoskeletal pain? A 4-year follow-up study in an industrial population. Int Arch Occup Environ Health.

[CR23] Neupane S, Leino-Arjas P, Nygård CH, Miranda H, Siukola A, Virtanen P (2015). Does the association between musculoskeletal pain and sickness absence due to musculoskeletal diagnoses depend on biomechanical working conditions?. Int Arch Occup Environ Health.

[CR24] Neupane S, Nygård CH, Oakman J (2016). Work-related determinants of multi-site musculoskeletal pain among employees in the health care sector. Work.

[CR25] Neupane S, Leino-Arjas P, Nygård CH, Oakman J, Virtanen P (2017). Developmental pathways of multisite musculoskeletal pain: what is the influence of physical and psychosocial working conditions?. Occup Environ Med.

[CR26] Neupane S, Nygård CH, Prakash KC, von Bonsdorff MB, von Bonsdorff ME, Seitsamo J, Rantanen T, Ilmarinen J, Leino-Arjas P (2018). Multisite musculoskeletal pain trajectories from midlife to old age: a 28-year follow-up of municipal employees. Occup Environ Med.

[CR27] Pan F, Byrne KS, Ramakrishnan R, Ferreira M, Dwyer T, Jones G (2019). Association between musculoskeletal pain at multiple sites and objectively measured physical activity and work capacity: results from UK Biobank study. J Sci Med Sport.

[CR28] Petersen J, Kirkeskov L, Hansen BB, Begtrup LM, Flachs EM, Boesen M, Hansen P, Bliddal H, Kryger AI (2019). Physical demand at work and sick leave due to low back pain: a cross-sectional study. BMJ Open.

[CR29] Prince SA, Adamo KB, Hamel ME, Hardt J, Connor Gorber S, Tremblay M (2008). A comparison of direct versus self-report measures for assessing physical activity in adults: a systematic review. Int J Behav Nutr Phys Act.

[CR30] Saastamoinen P, Leino-Arjas P, Laaksonen M, Martikainen P, Lahelma E (2006). Pain and health related functioning among middle-aged employees. J Epidemiol Community Health.

[CR31] Skotte J, Korshøj M, Kristiansen J, Hanisch C, Holtermann A (2014). Detection of physical activity types using triaxial accelerometers. J Phys Act Health.

[CR32] Solidaki E, Chatzi L, Bitsios P (2010). Work-related and psychological determinants of multisite musculoskeletal pain. Scand J Work Environ Health.

[CR33] Stemland I, Ingebrigtsen J, Christiansen CS, Jensen BR, Hanisch C, Skotte J, Holtermann A (2015). Validity of the Acti4 method for detection of physical activity types in free-living settings: comparison with video analysis. Ergonomics.

[CR34] Vos T, Abajobir AA, Abate KH, Abbafati C, Abbas KM, Abd-Allah F, GBD 2017 Disease and Injury Incidence and Prevalence Collaborators (2017). Global, regional, and national incidence, prevalence, and years lived with disability for 328 diseases and injuries for 195 countries, 1990–2016: a systematic analysis for the Global Burden of Disease Study 2016. The Lancet.

[CR35] World Health Organization (2003). The burden of musculoskeletal conditions at the start of the new millennium: report of a WHO Scientific group (WHO Technical Report Series: 919).

[CR36] Zochetti C, Consinni D, Bertazzi PA (1995). Estimation of prevalence rate ratios from cross-sectional data. Int J Epidemiol.

